# Antiretroviral Therapy Outcomes in HIV-Infected Children after Adjusting Protease Inhibitor Dosing during Tuberculosis Treatment

**DOI:** 10.1371/journal.pone.0017273

**Published:** 2011-02-23

**Authors:** Cordula Frohoff, Magendhree Moodley, Lee Fairlie, Ashraf Coovadia, Harry Moultrie, Louise Kuhn, Tammy Meyers

**Affiliations:** 1 Gertrude H. Sergievsky Center, College of Physicians and Surgeons, and Department of Epidemiology, Mailman School of Public Health, Columbia University, New York, New York, United States of America; 2 Wits Institute for Sexual Reproductive Health HIV & Related Diseases, Faculty of Health Sciences, University of the Witwatersrand, Johannesburg, South Africa; 3 Empilweni Services and Research Unit, Rahima Moosa Mother and Child Hospital, Department of Paediatrics, Faculty of Health Sciences, University of the Witwatersrand, Johannesburg, South Africa; 4 Harriet Shezi Clinic, Chris Hani Baragwanath Hospital, Department of Paediatrics, Faculty of Health Sciences, University of the Witwatersrand, Johannesburg, South Africa; University of Cape Town, South Africa

## Abstract

**Background:**

Modification of ritonavir-boosted lopinavir (LPV/r)-based antiretroviral therapy is required for HIV-infected children co-treated for tuberculosis (TB). We aimed to determine virologic and toxicity outcomes among TB/HIV co-treated children with the following modifications to their antiretroviral therapy (ART): (1) super-boosted LPV/r, (2) double-dose LPV/r or (3) ritonavir.

**Methods and Findings:**

A medical record review was conducted at two clinical sites in Johannesburg, South Africa. The records of children 6–24 months of age initiating LPV/r-based therapy were reviewed. Children co-treated for TB were categorized based on the modifications made to their ART regimen and were compared to children of the same age at each site not treated for TB.

Included are 526 children, 294 (56%) co-treated for TB. All co-treated children had more severe HIV disease, including lower CD4 percents and worse growth indicators, than comparisons.

Children in the super-boosted group (n = 156) were as likely to be virally suppressed (<400 copies/ml) at 6 months as comparisons (69.2% vs. 74.8%, p = 0.36). Children in the double-dose (n = 47) and ritonavir groups (n = 91) were significantly less likely to be virally suppressed at 6 months (53.1% and 49.3%) than comparisons (74.8% and 82.1%; p = 0.02 and p<0.0001, respectively). At 12 months only children in the ritonavir group still had lower rates of virological suppression relative to comparisons (63.9% vs 83.3% p<0.05). Grade 1 or greater ALT elevations were more common in the super-boosted (75%) than double-dose (54.6%) or ritonavir (33.9%) groups (p = 0.09 and p<0.0001) but grade 3/4 elevations were observed in 3 (13.6%) of the super-boosted, 7 (15.9%) of the double-dose and 5 (8.9%) of the ritonavir group (p = 0.81 and p = 0.29).

**Conclusion:**

Good short-term virologic outcomes were achieved in children co-treated for TB and HIV who received super-boosted LPV/r. Treatment limiting toxicity was rare. Strategies for increased dosing of LPV/r with TB treatment warrant further investigation.

## Introduction

An increasing proportion of the global burden of tuberculosis (TB) is related to HIV, with the highest rate of dual infection occurring in Sub-Saharan Africa.[1] In 2008, 9.4 million new TB cases were diagnosed globally of which 30% occurred in the African region.[2] Children make up approximately 10% of the TB burden.[3] One of a few African studies estimates the incidence of TB in HIV-infected children to be 1,600 per 100,000 increasing further in severely immunocompromised children or children with high viral load.[4] Compared with HIV-uninfected children with TB, HIV-infected children with TB and not receiving antiretroviral therapy (ART) have a six times greater risk of dying.[5]

Both TB and HIV require multiple drugs for effective treatment and the situation is complicated by drug interactions between antiretrovirals and rifampicin, the most commonly used drug for TB treatment. Rifampicin is a strong inducer of cytochrome p450 enzymes, particularly CYP3A isoenzymes and p-glycoprotein, [6], [7] resulting in accelerated clearance of protease inhibitors (PI).[8], [9], [10], [11] Ritonavir is a strong inhibitor of CYP3A.[12] Most PI's are boosted with low dose ritonavir to ensure sustained and elevated plasma levels. However, studies in adults have shown that the mininmum serum concentration (Cmin) of indinavir, lopinavir and atazanavir were decreased by >90% when given with the standard ritonavir-boosting dose of 100 mg in the presence of rifampicin.[8], [9], [10]

PI-based therapy using ritonavir-boosted lopinavir (LPV/r) is the recommended first-line regimen for HIV-infected infants and young children <3 years of age in South Africa,[13] and WHO recommends that infants failing nevirapine-containing prophylaxis should be started on a PI containing regimen.[14]

A pediatric study confirmed that trough concentrations of lopinavir with rifampicin were best preserved by adding extra ritonavir to give a LPV/r: Ritonavir ratio of 1∶1 referred to as “super-boosted” LPV/r.[15] However, while in the pediatric study, there were no treatment interruptions for liver enzyme elevations [15], super-boosted dosing has been associated with hepatotoxicity in adults.[10] A simpler approach would be to increase the LPV/r dose, although double-dosing LPV/r in HIV-infected children receiving rifampicin did not result in the achievement of therapeutic levels of lopinavir.[11] Combined use of lopinavir, ritonavir and rifampicin is challenging requiring evaluation of efficacy and toxicity.[16]

Here we evaluate, in the context of two large pediatric HIV treatment programs in Johannesburg, South Africa, the toxicity and therapeutic outcomes of ritonavir or adjusted dose regimens of LPV/r in combination with rifampicin-based TB treatment among HIV-infected children initiating ART between the ages of 6 months and 2 years.

## Methods

### Study sites

We conducted a retrospective record review at two clinical sites providing pediatric HIV treatment in Johannesburg, South Africa: the Harriet Shezi Children's Clinic (Shezi) at Chris Hani Baragwanath Hospital and the Neverest research site at Rahima Moosa Mother and Child Hospital. At both sites, infants and young children under the age of 2 years were initiated onto first-line therapy with LPV/r, lamivudine and stavudine. Children receiving TB treatment were compared to children of the same age receiving ART but no TB treatment, and co-treated children were further stratified by the modifications made to their PI. Virologic and toxicity outcomes within the first 6 to 12 months after ART and TB treatment in TB cases and ART initiation in the comparison group were investigated.

### Participants

The study population included all HIV-infected children 6–24 months of age who initiated ART between April 2004 and May 2007. The Shezi clinic provides HIV care for approximately 3 500 HIV-infected children. LPV/r, stavudine and lamivudine is the routine first-line regimen for children <3 years of age and clinically-stable children are followed at 3-monthly intervals. Viral loads, CD4 counts and liver function tests are conducted 6 monthly. The Neverest Study was a treatment strategies trial in which children who had prior exposure to NVP prophylaxis and subsequently required ART, were treated with LPV/r, stavudine and lamivudine. Enrollment started in April 2005. Once confirmed viral suppression occurred, children were randomized to continue LPV/r-based therapy or to start NVP-containing treatment. Viral loads and CD4 counts were conducted pre-treatment and three monthly thereafter. Doses were calculated based on body surface area calculations or weight and were modified at each visit.

At the time of the study, South African guidelines recommended ritonavir-based ART with TB treatment and this was adhered to for most of the Neverest study.[17] At Harriet Shezi Clinic it was the clinicians' decision to use adjusted doses of LPV/r. Initially super-boosted LPV/r was used, but double dosing of LPV/r was also used for some children because this was simpler for caregivers and children and adult data suggested feasibility of these approaches.[10]

### TB or BCG disease treatment

Treatment recommendations of pulmonary TB in children include rifampicin, isoniazid and pyrazinamide in the induction phase (2 months) and rifampicin and isoniazid in the continuation phase (4 months).[18] Extrapulmonary TB requires the addition of ethionamide or ethambutol in the induction phase and a longer course of treatment. BCG adenitis and BCG disease are treated with rifampicin, isoniazid and ethionamide or ethambutol for at least 6 months tailored to clinical response. Mycobacterium bovis is universally resistant to pyrazinamide. [13]

### Definition of the groups

Children were divided into those co-treated for TB (TB-treated) and those not treated for TB but who received LPV/r-containing ART (comparisons). If children were counted as TB-treated, their periods free of TB treatment were not included in the comparison cohort. Time0 in the TB-treated referred to the earliest time both rifampin-based treatment and ART were concomitantly received, irrespective of which was initiated first. Time0 in comparisons was the time of initiation of antiretroviral treatment. Given the high frequency of TB treatment in these cohorts, all children not receiving TB treatment were included as comparisons.

The basis on which the decision to initiate rifampicin-based TB treatment was extracted from the records. Clinical suspicion included any of the following; TB contact, cough of >2 weeks duration, failure to thrive and/or suggestive radiological findings. Definitive diagnosis included either a positive tuberculin skin test (≥5 mm induration),[17] a positive smear for acid fast bacilli or a positive culture for *Mycobacterium tuberculosis* or *Mycobacterium bovis*.

TB-treated children were subdivided into three groups according to the formulation of the PI that was received whilst receiving TB treatment namely:

1. Super-boosted LPV/r - 230 mg/m^2^ Lopinavir and ritonavir 57.5 mg/m^2^ with additional ritonavir 172.5 mg/m^2^
2. Double-dose LPV/r - 460–600 mg/m^2^
3. Ritonavir only - 460–600 mg/m^2^

Children who changed categories while being treated for TB were included in the category they spent the most time.

Children were excluded from the study if they had chronic liver disease, *Mycobacterium Avium Complex* infection or metabolic disease. They were also excluded if they received an initial regimen which included drugs other than LPV/r, ritonavir, stavudine and lamivudine, or if their paper record could not be found. Participants were considered lost to follow up if there was no 12 month visit and they were not known to have died by that point.

### Antiretroviral treatment outcomes

Toxicity was assessed by alanine aminotransferase (ALT) and hemoglobin measurement and whether or not treatment was interrupted. ALT and hemoglobin values were classified into their toxicity grade using normal values for this population from the local laboratory and Division of AIDS, National Institute of Health (DAIDS) grading tables.[19] ART outcomes included viral suppression to <400 copies/ml using the standard assay (quantification range 400–750,000 copies/ml, Roche Amplicor, Branchburg, NJ), CD4 response and changes in weight-for-age and height-for-age. Weight-for- age Z-scores (WAZ) and height-for-age Z-scores (HAZ) were calculated using WHO software.[20] Anthropometric measurements and laboratory data were extracted from the patients' files and the clinic databases.

### Ethics

The Neverest study was approved by the institutional review board of Columbia University and the Human Sciences Research Ethics Committee (HSREC) of the University of the Witwatersrand. Approval to review hospital record retrospectively at Harriet Shezi Children's Clinic was obtained from the HSREC of the University of the Witwatersrand. Informed consent was required for children participating in the Neverest study, although at Harriet Shezi, informed consent to store patient information in the data base was not obtained since this was originally conceived for primary care purposes.

### Statistical analysis

Clinical characteristics of the children pre-ART and within 6 and 12 months of time0 were compared between groups. TB-treated children receiving super-boosted and double-dose LPV/r at Shezi and TB-treated children on ritonavir from Neverest were compared to nonTB-treated children receiving LPV/r therapy at each site respectively (comparison groups). Wilcoxon tests were used to compare continuous and Chi-square test or Fisher's exact test for categorical variables. To consider whether pre-treatment differences between the groups explained differences in viral suppression rates, multivariable logistic regression models were conducted. Factors significantly associated with the outcome (p<0.05) or which once included modified the association between treatment group by >10% were included in final models.

## Results

### Study population

**Figure 1:**
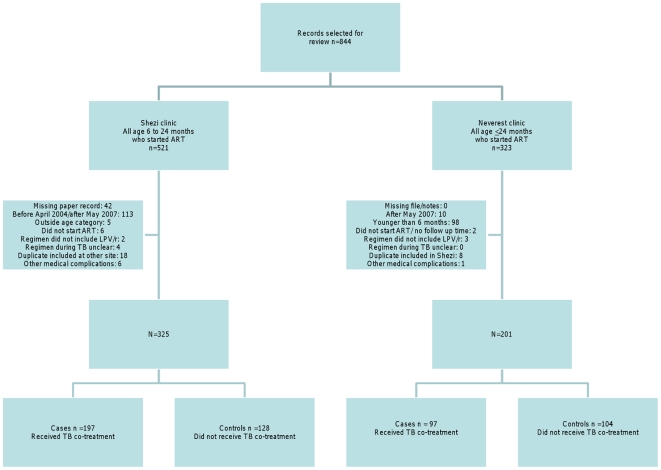
Flow diagram of study participants.

Between the two sites, 526 children 6–24 months were initiated onto a LPV/r-based regimen and 294 (56%) were co-treated for TB (figure 1). Of these, 156 (53.1%) received super- boosted LPV/r, 47 (16.0%) double-dose LPV/r and 91 (31.0%) ritonavir-based regimens. ART was started after TB treatment in 78.2% of TB-treated and before TB treatment in 21.8%. Amongst these, the median time between ART and TB treatment initiation was 54 days (IQR: 32-93) and did not differ between the three groups (Table 1). In children who started TB treatment after ART, the median time from ART start until TB treatment start was 42 days (IQR: 21.5-84). Most of the children (76.5%) who initiated TB treatment had a diagnosis of pulmonary TB and 8.4% of these were additionally diagnosed with BCG disease. Eight children (2.7%) were diagnosed with extra-pulmonary TB disease (2 meningitis, 4 abdominal, one TB adenitis and one unknown). A further 30 children received TB treatment only for BCG disease (Table 1). Children diagnosed with pulmonary TB were older (median age in months at ART start: 13 IQR: 10-17) than children diagnosed with BCG alone (median age of 7 months (IQR: 7-10); p<0.0001). TB was confirmed on microbiological grounds in 17.2% and PPD in 7.4%. Clinical suspicion for TB was the basis for diagnosis in 75.4% of children.

**Table 1:** Characteristics of children who initiated TB treatment by modification made to antiretroviral treatment (ART) regimen.

	Total	Super-boosted LPV/r	Double-dose LPV/r	Ritonavir	p-value
**TB-treated**	294(100)	156(100)	47(100)	91(100)	
Confirmed TB	72(24.6)	41(26.3)	12(25.5)	19(20.9)	
Clinical suspicion for TB	222(75.4)	115(73.7)	35(74.5)	72(79.1)	0.63
TB therapy at ART start	230 (77.6)	128 (82.1)	37 (78.7)	65 (71.4)	0.15
Median days (IQR) from TB therapy to ART start	54 (32–93)	56 (28–94)	46 (22–74)	55 (43–93)	0.18
Median days (IQR) ART start to TB therapy start	42 (21–84)	42 (29–80)	37(21–252)	41 (18–75)	0.89
**Type of TB diagnosis** *					
Pulmonary TB	225 (76.5)	128 (82.1)	35 (74.5)	62 (68.1)	0.04
Extra-pulmonary TB	8 (2.7)	5 (3.3)	1 (2.2)	2 (2.2)	0.86
BCG disease	49 (16.7)	27 (17.3)	7 (14.9)	15 (16.5)	0.93
Unknown	31 (10.5)	8 (5.1)	9 (19.1)	14 (15.4)	0.004
**Diagnostic evaluations** *					
*Pulmonary TB*					
Radiological-suspected	146 (64.9)	95 (74.2)	24 (68.6)	27 (43.6)	0.0002
Positive culture or smear	38 (16.9)	28 (21.9)	5 (13.9)	5 (8.2)	0.23
PPD positive	16 (7.1)	8 (6.3)	2 (5.7)	6 (9.8)	0.18
Other diagnostic test**	3 (1.3)	2 (1.6)	1 (2.9)	0 (0)	0.39
*Extra-pulmonary TB*					
Positive culture or smear	2(25)	2(40)	0(0)	0(0)	0.45
PPD positive	1(10)	0(0)	1(50)	0(0)	0.02
*BCG disease*					
Positive culture or smear	13(26.5)	6(22.2)	2(28.6)	5(33.3)	0.14
PPD positive	1(2.0)	0(0)	0(0)	1(6.7)	0.08
**Median days (IQR) duration of TB therapy**					
All TB-treated	203 (181–261)	200 (178–254)	198 (184–244)	208 (184–265)	0.30
Pulmonary TB	200 (178–238)	195 (176–236)	198 (175–221)	204 (182–258)	0.30
Extra-pulmonary TB	245 (195–263)	212 (180–245)	229 (195–263)	321 (279–363)	0.10
BCG disease	212 (184–271)	236 (183–299)	195 (192–205)	206 (196–253)	0.56
*Other medications* ***	9 (3.1)	6 (3.9)	1(2.1)	2 (2.2)	0.71

* Percents add up to >100% because more than one type of TB or diagnostic intervention was possible in the same child.

** Other diagnostic evaluations include lymph node biopsy (2), gastric washing (1).

*** Other medications include prednisone (3), ciprobay (3), ethambutol (3).

### Pre-ART characteristics

TB-treated children had significantly lower pre-ART CD4 percents and lower HAZ and WAZ than comparisons (Table 2). Severity of disease differed across the sites with more severe disease at the Shezi site. Pre-ART viral load, ALT and hemoglobin did not differ between the three co-treatment groups and their comparisons (Table 2). The median age at ART start in children co-treated for TB prior to initiating ART was 13.2 months (IQR: 9.7-17) compared to a median age of 9.2 months (IQR: 6.9-15.6 months) in co-treated children who started TB treatment after ART (p<0.0001).

**Table 2:** Pre-antiretroviral treatment (ART) characteristics stratified by the modification made to the ART regimen among 294 children treated for TB and 232 comparison children not treated for TB at each site.

	Super-Boosted LPV/r (n = 156)	Double dose LPV/r (n = 47)	Comparisons Shezi (n = 128)	Ritonavir (n = 91)	Comparisons Neverest (n = 104)
**Sex** a					
Male	72 (46.8)	28 (59.6)	54 (42.2)	53 (58.2)	55 (52.9)
Female	82 (53.2)	19 (40.4)^c^	73 (57.5)	38 (41.8)	49 (47.1)
**Age at ART start**					
Median (IQR) age inmonths	12.99(9.34–16.82) b	13.22(9.54–17.04)	14.82(9.69–19.42)	11.25(8.45–15.66)	11.43(7.96–16.46)
<12 mos (%)	70(44.9)	21(44.7)	50(39.1)	48(52.8)	57(54.8)
12-<18 mos (%)	51(32.7)	17(36.2)	34(26.6)	29(31.9)	29(27.9)
18–24 mos (%)	35(22.4)	9(19.2)	44(34.4)	14(15.4)	18(17.3)
**CD4 count in cells/ml**Median (IQR)	509(221–847) b	364(165–740) c	786(456–1197)	661(271–1079) d	927(636–1311)
**CD4 percent**Median (IQR)	10.7(7.1–16.8) b	9.51(4.96–16.7) c	13.8(10.5–18.6)	13.8(9.26–18.5) d	18.7(13.6–23)
**CD4 percent**					
<15%	106 (69.3)	32 (71.1)	72 (58.1)	51 (61.5)	30 (30.6)
15-<25%	32 (20.9)	12 (26.7)	33 (26.6)	22 (26.5)	49 (50)
≥25%	15 (9.8)	1 (2.2)	19 (15.3)	10 (12.1) d	19 (19.4)
Total	153	45	124	83	98
**HIV RNA in copies/ml (%)**					
<100,000	24 (17.1)	5 (11.4)	27 (22.3)	8 (9.4)	11 (11.6)
100,000–749,999	55 (39.3)	15 (34.1)	45 (37.2)	26 (30.6)	29 (30.5)
≥750,000	61 (43.6)	24 (54.6)	49 (40.5)	51 (60)	55 (57.9)
Total	140	44	121	85	95
**Mean (SD) WAZ-score**	−3.28 (1.73) b	−3.11 (1.93) c	−2.34 (1.70)	−2.70 (1.66) d	−2.07 (1.68)
**Mean (SD) HAZ-score**	−3.27 (1.60) b	−3.17 (2.14) c	−2.62 (1.53)	−3.64 (1.56) d	−3.14 (1.66)

a Denominators in each group are as shown.

b superboosted LPV/r group significantly different from Shezi comparisons p<0.05.

c double dose LPV/r group significantly different from Shezi comparisons p<0.05.

d ritonavir group significantly different from Neverest comparisons p<0.05.

### Mortality and loss to follow-up

In the 12 months after time0, 46 (8.9%) children died. This includes 17/232 (7.3%) comparisons, 21/230 (9.1%) co-treated for TB at time of ART initiation and 8/64 (12.5%) who initiated TB treatment after ART, there were no significant differences in mortality between the TB-treated and comparison groups. In the super-boosted LPV/r group 17 (10.9%) died, in the double-dose, one child (2.1%), and in the Ritonavir group, 11 children (12.1%) died. Children who died were significantly younger (median age at ART initiation 10 months (IQR: 7-15) than children who survived (median age 13 months, IQR: 9-18, p = 0.0008). Children who died had lower mean WAZ, higher pre-treatment viral loads and lower CD4 percentages (data not shown).

In the 12 months following time0, 45 children (8.5%) were lost to follow-up and another 22 children (4.2%) were transferred to other services and did not have 12 months data available. There were no significant differences between the groups in rates of loss to follow-up.

### Toxicity and treatment interruptions in co-treated children

Among the children assessed for liver function, ALT elevations ≥grade 1 were more common in the super-boosted group (75%) than in the double-dose (54.6%) or ritonavir (33.9%) groups (p = 0.09 and p<0.0001) but differences did not reach significance if only elevations greater than grade 2 were considered (Table 3). Seventeen children (5.8%) had an ART interruption while co-treated for TB: 6 (3.9%) in the super-boosted, 3 (6.4%) in the double-dose and 8 (8.8%) in the Ritonavir group (p = 0.24). One child in the super-boosted group and 3 children in the ritonavir group interrupted treatment because of an elevated ALT and one in the ritonavir group due to neutropenia. Among the comparisons, 5 (3.9%) at the Shezi site and 7 (6.7%) at the Neverest site interrupted ART. One of the interruptions among the comparisons was due to hepatotoxicity with an ALT grade 4. Treatment interruptions were most commonly due to self-reported missed doses for reasons unrelated to toxicity and did not differ significantly between the groups. TB treatment was interrupted in 13 (8.6%) children in the super-boosted group, three (6.5%) in the double-dose, and two (2.6%) in the Ritonavir group. Two children interrupted TB treatment due to hepatotoxicity (one in the RTV and one in the double-dose group). When comparing ALT levels measured 12 months after time0 there were no differences between the groups.

**Table 3:** Toxicity outcomes during 12 months after initiation of TB co-treatment by modification made to the PI-containing regimen.

	Super-Boosted LPV/r	Double dose LPV/r	Ritonavir
In follow up at last assessment	118 (75.6)	34 (72.3)	74 (81.3)
Died within 12 months	17(10.9)	1(2.1)	11(12.1)
**Highest ALT while co treated for TB**			
Grade 0 (<38 IU/ml)	11(25)^a^	10(45.5)	37(66.1)
Grade 1 (38–75 IU/ml)	20(45.5)	7(31.8)	10(17.9)
Grade 2 (76–150 IU/ml)	6(13.6)	2(9.1)	4(7.1)
Grade 3 (151–300 IU/ml)	4(9.1)	2(9.1)	2(3.6)
Grade 4>300 IU/ml	3(6.8)	1(4.6)	3(5.4)
**Lowest Hemoglobin while co-treated for TB**			
Grade 0 (>10 g/dl)	15(79.0)^a^	7(63.6)	18(48.7)
Grade 1 (8.5–10.0 g/dl)	3(15.8)	3(27.3)	13(35.1)
Grade 2 (7.5–8.4 g/dl)	1(5.3)	1(9.1)	4(10.8)
Grade 3 (6.5–7.4 g/dl)	0(0)	0(0)	1(2.7)
Grade 4 (<6.5 g/dl)	0(0)	0(0)	1(2.7)
**ART interrupted**	6(3.9)	3(6.4)	8(8.8)
**TB treatment interrupted**	13(8.6)	3(6.5)	2(2.6)

a Toxicity outcomes in children on superboosted LPV/r significantly different to children taking ritonavir only if ≥ grade 1 is selected as the cut-off.

### Virologic, clinical and immunological response

The proportions of children who achieved viral load <400 copies/ml by 6 months in the super-boosted LPV/r group (69.2%) did not differ significantly from the comparison group (74.8%; p = 0.36). Children in the double-dose and ritonavir groups were significantly less likely to be virally suppressed at 6 months (53.1% and 49.3%) than comparisons (74.8% and 82.1%; p = 0.02 and p<0.0001, respectively). At 12 months after time0, only children in the ritonavir group still had lower rates of virological suppression relative to comparisons (Table 4). After adjusting for pre-treatment CD4 percent and HAZ, the reduction in viral suppression at 6 months associated with double-dose LPV/r was attenuated and no longer significant (OR = 0.703; 95% CI: 0.251–1.966) but the association with ritonavir remained after adjusting for HAZ, ALT and hemoglobin (OR = 0.240; 95% CI: 0.109–0.527). During follow-up fourteen children (4.8%) had one additional modification to their ART regimen due to referral to clinical sites with different policies. These children were classified based on the regimen they received for the longest duration. Excluding these children from the analysis had no impact on any differences in responses observed between the groups.

**Table 4:** Virologic, clinical and immunological outcomes at 6 and 12 months between children co-treated for TB stratified by the modifications made to their PI-based regimen and comparisons not co-treated for TB.

	Super-Boosted LPV/r	Double dose LPV/r	Comparisons Shezi	Ritonavir	Comparisons Neverest
**6 months after time 0**					
N	120	32	103	75	84
Median (IQR) CD4 count	1265 (880–1712)	1084 (54–1609) b	1375 (1025–1797)	1370 (1007–2166)^c^	1723 (1258–2485)
Median (IQR) CD4%	20.8 (15.9–27.4)^a^	19.5 (12.7–26.6) b	24.7 (18.9–30.5)	23.9 (16.6–30.0) c	29.1 (20.0–33.2)
CD4%<15	26(21.7)	12(37.5)	14(14.4)	14(20)	5(6.2)
CD4% 15–24.9	57(47.5)	8(25.0)	36(37.1)	26(37.1)	28(34.6)
CD4%≥25	37(30.8)^a^	12(37.5) b	47(48.5)	30(42.9) c	48(59.3)
Median CD4% change (IQR)	8.60 (3.2–13.80)	7.08 (0.2–14.0)	9.17 (5.39–13.19)	9.30 (4.40–14.9)	9.3 (3.5–13.8)
VL<400 c/ml	81(69.2)	17(53.1)	77(74.8)	37(49.3)	69(82.1)
VL≥400 c/ml	36(30.8)	15(46.9) b	26(25.2)	38(50.7) c	15(17.9)
Mean (SD) WAZ -score	−1.75(1.34) a	−1.93(1.79) b	−1.00(1.24)	−1.26(1.56)	−0.94(1.24)
Mean WAZ change	1.58(1.25) a	1.28(1.40)	1.19(1.00)	1.44(1.11)	1.11(1.14)
Mean (SD) HAZ score	−2.93(1.36)	−3.11(1.79)	−2.59(1.31)	−3.71(1.57)	−3.27(1.58)
Mean HAZ score change	0.53(1.24) a	0.48(1.75)	−0.016(1.28)	0.043(1.68)	−0.24(1.44)
**12 months after time0**					
N	106	27	84	61	36
Median (IQR) CD4%	26.0 (20.1–33.2)	25.0(15.2–33.1)	26.6 (21.8–32.3)	27.4(21.0–32.5) c	30.8(27.3–33.5)
CD4%<15%	15(13.0)	6(20.7)	5(5.4)	3(5.4)	0(0)
CD4% 15-<25%	32(30.2)	7(25.9)	31(37.4)	21(38.2)	3(10.7)
CD4%≥25%	59(55.7)	14(51.9) b	47(56.6)	31(56.4) c	25(89.3)
Median CD4% change	13.6(4.8–19.6)	11.8 (7.2–19.4)	11.5(7.2–17.9)	13.9(8.4–20.6)	13.3(7.1–19.4)
VL<400 c/ml	87(82.9)	20(76.9)	70(83.3)	39(63.9)	30(83.3)
VL≥400 c/ml	18(17.1)	6(23.1)	14(16.7)	22(36.1) c	6(16.7)
Mean (SD) WAZ-score	−1.16(1.24) a	−1.06(1.35)	−0.77(1.08)	−0.57(1.33)	−0.37(1.04)
mean WAZ change	2.19(1.53) a	2.06(1.77)	1.34(1.21)	2.08(1.27) c	1.34(1.45)
Mean (SD) HAZ-score	−2.69(1.36) a	−2.60(1.73)	−2.32(1.11)	−3.21(1.44)	−3.04(1.38)
mean HAZ score change	0.81(1.48)^a^	0.98(1.89)	0.31(1.20)	0.43(1.64)	0.08(1.88)

a super-boosted LPV/r group significantly different from comparisons p<0.05,

b double dose LPV/r group significantly different from comparisons p<0.05.

c ritonavir group significantly different from Neverest comparisons.

CD4 percentages in the first 6 months were significantly lower in all TB co-treatment groups compared to comparisons but this was due to lower pre-treatment values. Changes in CD4 percentage were similar regardless of group. The median increase in CD4% during the first 6 months of ART was 8.6% [IQR: 3.2-13.8] in the super- boosted group, 7.1% [IQR: 0.2-14.0] in the double-dose, and 9.3% [IQR: 4.4-14.9] in the ritonavir group. In the comparisons, the median CD4% increase was 9.2% [IQR: 5.4-13.2] and 9.3% [IQR: 3.5-13.8] (Table 4).

Overall WAZ increased from −2.73±1.8 at time 0 to −1.34±1.4 at 6 months and to −0.83±1.2 at 12 months. WAZ was lower in the super-boosted and double-dose groups relative to their comparisons at 6 months (p = 0.004 and p<0.0001) but there was no significant difference between the ritonavir group and the control group. At 12 months, the super-boosted group continued to have lower WAZ than the comparison group (−1.16±1.2 vs. −0.77±1.1, p = 0.01). From 0–6 months and from 0–12 months after time 0, the mean WAZ change was highest in the super-boosted group (WAZ change 1.58±1.3 vs. 1.1 9±1.0 in the comparisons; p = 0.005 and 2.19±1.5 vs. 1.34±1.2; p<0.0001, while the change from 0–6 months was not significantly different between the other two TB co-treatment groups and their comparisons (Table 4).

## Discussion

In high HIV prevalence areas where the burden of TB co-infection is also large, it is important to understand treatment responses of children to ART and rifampin-based co-treatment. During the South African ART rollout program beginning in April 2004, clinical practices for children co-treated with rifampicin and PI changed from switching to ritonavir, to super-boosting and double dosing LPV/r. This study provides data on virological, clinical and toxicity outcomes in children under 2 years who received one of three different modifications to their PI-based regimen whilst being co-treated with rifampicin-based TB therapy compared to comparison children not treated for TB.

Children who received a ritonavir-based regimen together with TB treatment were less likely to be virally suppressed at 6 months than those receiving super-boosted LPV/r and the comparison groups. In this group, poor virological outcomes may be explained by increased resistance mutations developing when ritonavir alone is used as part of combination ART.[21] Significant reductions in ritonavir levels have also been described when used with rifampicin and this might also explain the subsequent treatment failure seen in this group of children.[22] These findings confirm our prior results that ritonavir used alone in combination ART is a poor choice,[23] particularly in children co-treated with rifampicin, and should be avoided.

Virological suppression was similar in the super-boosted group and their comparisons. Our findings are consistent with a pediatric pharmacokinetic study which demonstrated that adding extra ritonavir to ‘super-boost’ LPV results in therapeutic drug levels of LPV.[24] However, super-boosting LPV/r has practical barriers and adds complexity to pediatric ART for the children, care-providers and for those maintaining stock control, since ritonavir is poorly palatable and has a short shelf-life. Clinicians, on occasion, elected the simpler approach of doubling the LPV/r dose. Pharmacokinetic studies have now suggested that this may result in an inadequate dose of LPV when used with rifampicin.[11] Our data showed that although viral suppression rates at six months were lower in those who received double dose LPV/r compared to super-boosted or comparisons, interestingly, by the 12 months viral load assessment, both groups had similar viral suppression rates to comparisons. Since this approach has many practical advantages, further evaluation of the double-dose approach, possibly at higher or more frequent doses, is required.

Ritonavir and the antituberculous drugs, particularly rifampicin and isoniazid have the potential to cause severe liver toxicity that could potentiate each other. Adult studies have shown that superboosting PI's may result in excess toxicity[10] and the Center for Disease Control and Prevention (CDC) cautions against TB co-treatment with super-boosted PI's.[25] In our cohort, there were no significant differences in the proportions of children with grade 3/4 ALT elevations in the TB co-treatment groups whilst receiving TB treatment compared to children on LPV/r alone. There were also only few treatment interruptions due to toxicity. This suggests that the use of boosted LPV/r and TB treatment in this group was generally well tolerated. After completion of TB therapy, there was no undue liver function derangement among those who had previously been on co-treatment for TB and HIV. Mortality rates were similar between the TB-treated and comparison groups, despite the fact that the children who received TB treatment were generally sicker and younger than the comparisons. The non-significant differences within the TB-treated groups is probably a chance occurrence due to small numbers, since it is unlikely that the differences in treatment modality would have had a protective effect. It is possible that some children in the comparison groups had undiagnosed TB or other opportunistic infections that increased their risk of death after commencing ART.

Currently there is no good alternative to rifampin-containing TB regimens for first-line TB treatment. A regimen without rifampicin is likely to result in delayed sputum conversion, prolonged duration of therapy and a higher incidence of relapse.[26], [27] In this class of drugs rifampicin is the only available option for the treatment of children. Rifabutin, which has been recommended for use with PI-containing regimens for adults [25] has been unavailable in resource-poor settings and there is no suitable rifabutin dosing and formulation for children.

There are limited ART options for infants who also have TB. Efavirenz is recommended for older children who require TB treatment and there does not appear to be significant interaction with rifampicin.[28] Efavirenz dosing in children <3 years of age is yet to be determined. WHO recommends that NVP at maximal dose or triple NRTI regimens be used in children under 3 years of age when TB treatment is required.[29] Despite reassurance from a trial that triple NRTI regimens have clinical benefit in adults,[30] there was poorer potency in terms of virological and immunlogical outcomes.[31] This option may therefore not be ideal for infants in whom the viral load is usually very high. There have been reports of adequate treatment responses with no excess toxicity when nevirapine-containing regimens have been used together with TB treatment,[30], [32] although rifampicin enhances nevirapine metabolism, leading to a risk of subtherapeutic concentration.[33] Additionally, concerns about emergence of nevirapine resistance following nevirapine prophylaxis have resulted in revision of WHO guidelines which now recommend PI-based therapy for infants starting ART who have failed nevirapine-containing prophylaxis.[14] Results from a multi-center trial confirm that, in the face of prior nevirapine exposure, LPV/r is superior for treatment of infants.[34]

Despite concerns about excess toxicity in adults, in infants and young children requiring ART and TB treatment who were assessed for liver function during the course of co-treatment, boosted LPV/r appears a safe and efficacious option for therapy. Increasing the dose of LPV/r with TB treatment, appears a safe alternative worth further exploration, albeit at higher or more frequent doses. Modeling has demonstrated that increasing LPV/r dosing by 2.5–5 times if given 12 hourly or decreasing the interval between doses to 8 hourly when used with rifampicin-containing TB therapy may result in therapeutic levels of lopinavir.[35] Ongoing pharmacovigilance is warranted since our retrospective data have limitations. The quest for new ART and TB formulations that are potent, easy to prescribe and tolerable when used together must urgently be pursued.
